# Effects of Al Addition on Microstructures and Mechanical Properties of CoCrFeMnNiAl*_x_* High Entropy Alloy Films

**DOI:** 10.3390/e22010002

**Published:** 2019-12-18

**Authors:** Ya-Chu Hsu, Chia-Lin Li, Chun-Hway Hsueh

**Affiliations:** Department of Materials Science and Engineering, National Taiwan University, Taipei 10617, Taiwan; r06527032@ntu.edu.tw (Y.-C.H.); chialinli@ntu.edu.tw (C.-L.L.)

**Keywords:** high-entropy alloy films, Al addition, microstructure, mechanical properties, phase transition, nanotwins

## Abstract

CoCrFeMnNiAl*_x_* (*x* = 0, 0.07, 0.3, 0.6, 1.0, 1.3) high-entropy alloy films (HEAFs) were processed by co-sputtering of CoCrFeMnNi alloy and Al targets. The effects of Al content on the microstructures and mechanical properties of HEAFs were studied. The XRD results indicated that the crystalline structure changed from the single face-centered cubic (FCC) phase for *x* = 0 and 0.07 to duplex FCC + body-centered cubic (BCC) phases for *x* = 0.3 and 0.6, and eventually, to a single BCC phase for *x* = 1.0 and 1.3, which agreed with the corresponding selected-area electron diffraction patterns. Also, nanotwins were observed in the FCC phase. Mechanical properties of films were studied using nanoindentation and micropillar compression tests. The hardness increased from 5.71 GPa at *x* = 0 to 8.74 GPa at *x* = 1.3. The compressive yield strength increased from 1.59 GPa to 3.73 GPa; however, the fracture strain decreased from 20.91% (no fracture) to 13.78% with the increasing Al content. Both nanotwins and BCC phase contributed to the strengthening effects for CoCrFeMnNiAl*_x_* HEAFs. Also, compared to the bulk CoCrFeMnNiAl*_x_* counterpart, the film exhibited much higher hardness and strength because of the much smaller grain size and the presence of nanotwins.

## 1. Introduction

High-entropy alloys (HEAs) are the mixture of five or more elements with nearly equiatomic compositions and possess a single solid solution phase rather than intermetallic compounds due to a high entropy effect [1,2,3]. HEAs have many promising properties such as good thermal stability, distinct electrical properties as well as excellent corrosion and wear resistances [4,5,6,7,8,9], and a recent review of HEAs was performed by Zhang et al. [10]. Taking the equiatomic CoCrFeMnNi alloy as an example, it is an FCC crystalline structure [3,11] and has been proven to be an ultra-ductile but low-strength alloy [12,13,14]. Also, it demonstrates exceptional mechanical properties and fracture toughness at cryogenic temperatures; however, its strength is low at room temperature [12,14,15].

To improve the strength and hardness of CoCrFeMnNi HEA, researchers have added a moderate amount of Al to modify these properties [16,17,18]. The crystalline structure changes from a single FCC phase to the duplex FCC + BCC phases, and eventually, to the single BCC phase. In this case, Al is a BCC inducer and there is a phase transition from FCC to BCC with the increasing Al content. The atomic radius of Al is relatively large (*r*_Al_ = 0.143 nm) compared to other constituent elements (*r*_Co_ = 0.125, *r*_Cr_ = 0.128, *r*_Fe_ = 0.126, *r*_Mn_ = 0.127 and *r*_Ni_ = 0.124 nm). The atomic radii of these constituent elements (Co, Cr, Fe, Mn and Ni) are very similar, and the effect of the atomic size difference in CoCrFeMnNi is small (~1.18%) [19]. However, the atomic size difference becomes larger with the addition of Al into the CoCrFeMnNi alloy system. Because the lattice distortion is determined by the atomic size difference, the lattice distortion can be observed and leads to the formation of a new BCC phase with the increasing Al content [17]. CoCrFeNiAl*_x_* [20,21,22,23], CrCuFeNiAl*_x_* [24], CoCrCuFeNiAl*_x_* [25,26,27], CoCrFeNiTiAl*_x_* [28], CoCrFeMnNiAl*_x_* [16,17,18] and (TiVCrMnFeCoNiCu)_100–*x*_Al*_x_* [29] systems have been studied previously, and the phase transformation and microstructural evolution with various Al contents have been shown. Specifically, the AlNi spherical particles were observed to precipitate in the matrix because the mixing enthalpy of AlNi was minimum and Al could be easily bonded with other constituent elements to form the binary compound.

Xian et al. [17] studied the effects of Al addition on the mechanical properties of CoCrFeMnNiAl*_x_* (*x* in molar ratio) HEAs in compression. The yield strength and hardness of CoCrFeMnNi HEA were 188.04 MPa and HV 163.55 (1.604 GPa), respectively. Both values increased dramatically with the addition of Al and had a maximum yield strength of 1.194 GPa for CoCrFeMnNiAl_0.75_ and hardness of HV 497.1 (4.875 GPa) for CoCrFeMnNiAl. Kim et al. [30] studied the effects of Al addition on the high-temperature deformation mechanisms of CoCrFeMnNiAl_0.5_ HEA and found that the hard BCC phase provided nucleation sites for dynamically recrystallized grains in the soft FCC matrix through particle-stimulated nucleation. Liu et al. [31] studied the effects of Al addition on microstructures and properties of CoCrCuFeNiAl*_x_* HEAs and found not only the enhanced friction and wear resistance but also the improved corrosion behavior because of the formation of a passivation layer in the presence of Al. Serrated deformation of HEAs has also been studied [32,33,34]. While the serrated plastic flow is attributed to shear-banding dynamics in amorphous bulk metallic glasses [35,36,37], serrations are typically caused by the locking of dislocations by solute atoms in crystalline materials [32,33,34]. Combining the high entropy effect with the amorphous structure, the high-entropy bulk metallic glasses have been developed which possess characteristics of metallic glasses with small sample sizes, characteristics of HEAs for atomic composition, and unique properties from both HEAs and bulk metallic glasses [38].

In this study, a series of CoCrFeMnNiAl*_x_* (*x* = 0–1.3) high-entropy alloy films (HEAFs) were deposited by radio frequency (RF) magnetron co-sputtering. The effects of Al addition on the microstructures and mechanical properties of CoCrFeMnNiAl*_x_* HEAFs were systematically studied. The correlation between the phase transition and mechanical properties at various Al contents was investigated, and the differences between CoCrFeMnNiAl*_x_* HEAs and HEAFs were compared.

## 2. Materials and Methods

CoCrFeMnNiAl*_x_* HEAFs were prepared by co-sputtering of CoCrFeMnNi alloy and Al targets with the purity of each element higher than 99.9 wt.%. Sputtering is effective in depositing alloy films from a single target even though the individual elements have different sputter yields, because the surface composition of the target equilibrates after a pre-sputter step. The Si wafer was chosen as the substrate and cleaned sequentially in acetone, ethanol and deionized water for 15 min in each step before the film deposition. The CoCrFeMnNi alloy and Al targets were pre-sputtered for 15 min each to remove the contaminated surface and to equilibrate the surface composition of the alloy target. CoCrFeMnNiAl*_x_* films were deposited by the RF magnetron sputtering system with a fixed power of 400 W on a CoCrFeMnNi target. The power on Al target was varied from 0 to 80 W to control the content of Al in the film. The base pressure was better than 2.8 × 10^–4^ Pa. The working distance, working pressure and Ar flow were maintained at 10 cm, 0.4 Pa and 20 sccm, respectively. The deposition time was 1.5 h and the film thickness increased from 2.47 to 3.41 μm when the power applied to the Al target increased from 0 to 80 W. The substrate was rotated at 14 rpm to ensure the homogeneity of the as-deposited CoCrFeMnNiAl*_x_* films during the film deposition.

The compositions of the films were determined by the electron probe X-ray microanalyzer (EPMA, JEOL JXA-8200). The crystalline structures were examined by X-ray diffraction (XRD, Rigaku TTRAX 3) with Cu K_α_ (*λ* = 0.15406 nm) radiation. The scanning angle, 2*θ*, ranged from 20° to 100°. The surface morphology and film thickness were analyzed using a field-emission scanning electron microscope (SEM, NOVA NANO SEM 450) with 10 kV of accelerate voltage.

The hardness was measured by Hysitron TI 950 TriboIndenter (Bruker, Minneapolis, MN, USA) using a Berkovich indenter with a tip radius of 150 nm. Calibration of this instrument was routinely performed with a standard fused quartz specimen (*H* = 9.25% ± 10% GPa, *E_r_* = 69.6% ± 5% GPa). For each film, nine indentations were performed with the space between indentation sites greater than 10 μm, and the average of the data without the minimum and the maximum was used as the hardness of the film. A dual-beam focused ion beam (FIB, FEI Quanta 3D FEG) was used to prepare the micropillars and TEM samples. Measurements of mechanical properties were performed using in-situ SEM micropillar compression tests (Hysitron PI 85 SEM PicoIndenter) with a flat punch diameter of 5 μm. All compression experiments were conducted at room temperature and the strain rate was 10^–3^ s^–1^. The aspect ratios of pillars ranged from 2.6 to 3.2 and the taper angles were 2° to 5°. Finally, the transmission electron microscope (TEM, FEI Tecnai G2 F20) was used to observe the detailed microstructure and reconfirm the phase structure.

## 3. Results and Discussion

The elemental compositions (at.%) of the CoCrFeMnNi alloy target were 20.14% ± 1.07% of Co, 19.64% ± 0.90% of Cr, 19.88% ± 1.43% of Fe, 21.95% ± 0.65% of Mn, and 18.39% ± 0.78% of Ni. The contents of Co, Cr, Fe, Mn and Ni were approximately equal to the nominal equiatomic percentage of 20 at.%.

### 3.1. Compositions and Crystalline Structure of CoCrFeMnNiAl_x_ HEAFs

CoCrFeMnNiAl*_x_* (*x* = 0, 0.07, 0.3, 0.6, 1.0, 1.3, denoted as Al*_x_* hereafter) HEAFs were deposited by RF sputtering. The powers applied on the Al target, molar ratios and the chemical compositions of the films, measured by EPMA and averaged from seven measurements for each film, are summarized in Table 1. The content of each element in Al_0_ was slightly different from that of the CoCrFeMnNi alloy target and the near-equiatomic CoCrFeMnNi HEAF was achieved. The content of Al in the film increased with the increasing power applied on the Al target.

**Figure 1:**
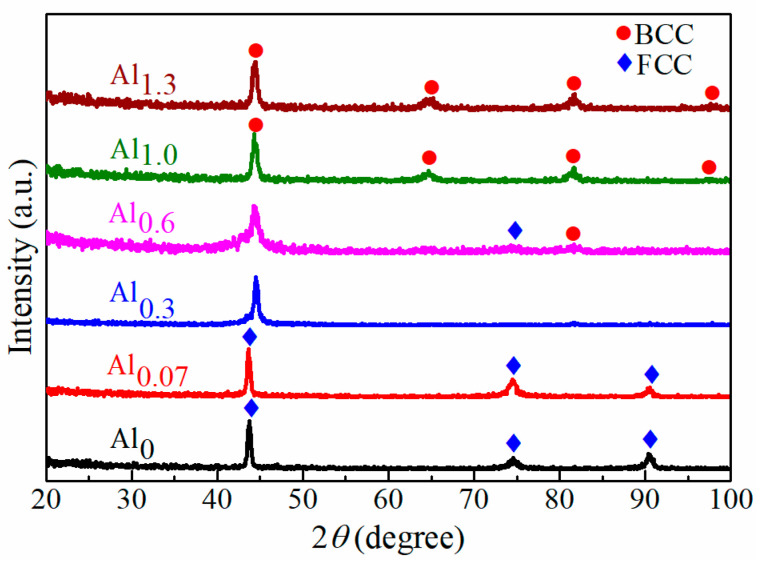
XRD patterns of CoCrFeMnNiAl*_x_* HEAFs with a scan rate of 4°/min.

**Figure 2:**
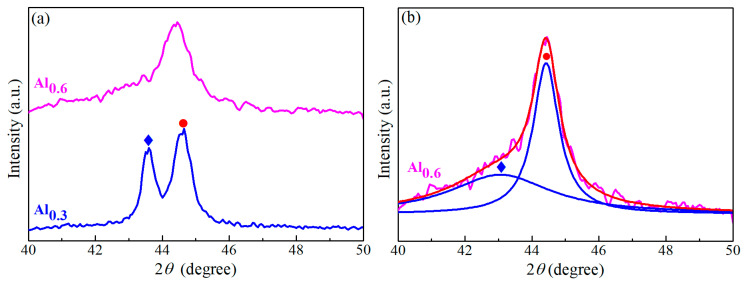
(**a**) XRD patterns of Al_0.3_ and Al_0.6_ using a slower scan rate of 1°/min and (**b**) the deconvoluted pattern for Al_0.6_ from (a).

With the scan rate of 4°/min, the XRD patterns of CoCrFeMnNiAl*_x_* films with different Al contents are shown in Figure 1. To obtain a better resolution for Al_0.3_ and Al_0.6_ samples, the slower scan rate of 1°/min was used from 40° to 50° and the corresponding XRD patterns are shown in Figure 2a. In addition, the deconvoluted pattern of Al_0.6_ is shown in Figure 2b. The diffraction peaks located at around 43.6°, 74.5° and 90.5° in 2*θ* were identified as the (111), (220) and (311) planes of the FCC structure, and the diffraction peaks at around 44.3°, 64.7°, 81.6° and 97.7° were assigned to the (110), (200), (211) and (220) planes of the BCC crystal. As observed, all the diffraction peaks for Al_0_ and Al_0.07_ films were identified to be a single FCC structure, indicating that only a single FCC solid solution was formed. The (110)_BCC_ diffraction peak appeared at around 44.3° in Figure 2a when the Al content was increased to 6.04 at.% (Al_0.3_), suggesting the formation of a new crystalline structure of BCC by adding a moderate amount of Al. Then, the relative intensity of the (110)_BCC_ diffraction peak increased and that of (111)_FCC_ peak decreased with the increasing Al content, as shown in Figure 1. With further addition of Al, all FCC diffraction peaks disappeared and only the BCC peaks could be identified in Al_1.0_ and Al_1.3_. Therefore, it could be concluded from Figure 1 and Figure 2 that the phase transformation from FCC to BCC occurred with the increasing Al content, and the higher Al contents tended to stabilize the BCC structure. Compared to the published results of CoCrFeNiAl_0.3_ HEAF exhibiting only a single FCC phase [39,40], the present work of CoCrFeMnNiAl_0.3_ HEAF showed the duplex FCC + BCC phases. This suggested that the presence of Mn could reduce the stability of FCC and favor the formation of BCC, and it agreed with other published research [41].

**Figure 3:**
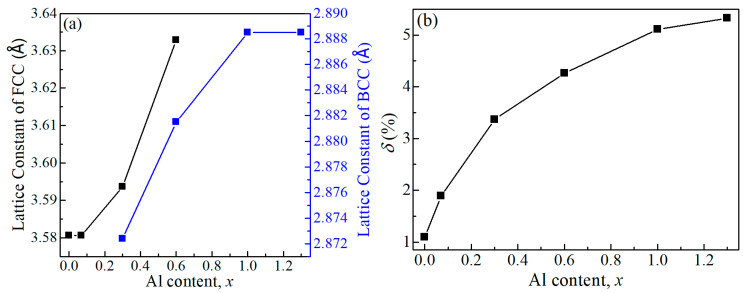
(**a**) Lattice constants of FCC and BCC structures and (**b**) the atomic size difference (*δ*) of CoCrFeMnNiAl*_x_* HEAFs as functions of Al content.

The lattice constants of FCC and BCC structures as functions of the Al content are shown in Figure 3a, and both increased with the increasing Al content because of the larger atomic radius of Al compared to other constituent elements. The atomic size difference (*δ*) is a parameter to describe the lattice distortion and structural instability, and it is defined by [42,43]

(1) $$\delta =100\cdot \sqrt{\sum_{i=1}^{n}c_{i}\;{\left(1-r_{i}/\bar{r}\right)}^{2}},$$

where *n* is the number of constituent elements in the system, *c_i_* is the atomic percentage of the *i*th element, *r_i_* is the atomic radius of the *i*th element and $\bar{r}=\sum_{i=1}^{n}c_{i\;}r_{i}$ is the average atomic radius.

The dependence of *δ* on the Al content is presented in Figure 3b. In the absence of Al, *δ* is ~1.1%. With the addition of Al, the value of *δ* increases from 1.1% to 5.33%. Apparently, the large size mismatch among these atoms would lead to the lattice distortion and the structural instability; i.e., the formation of BCC structure could be attributed to the larger atomic size difference between Al and other constituent elements. As a result, the crystalline structure changes from a closely packed FCC to a loosely packed BCC structure.

### 3.2. Surface Morphology and Microstructure

**Figure 4:**
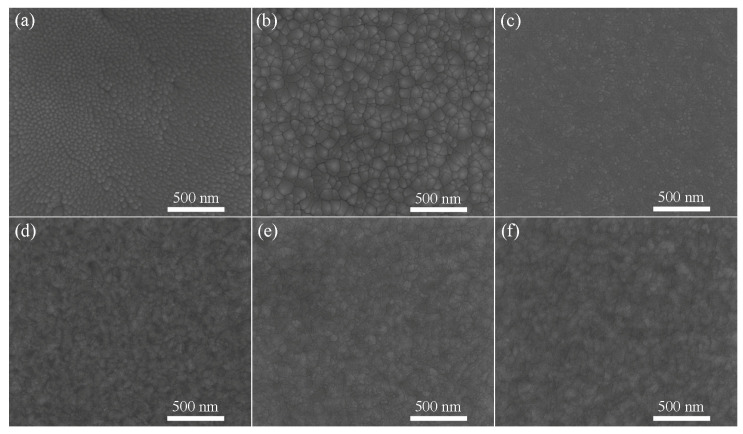
SEM micrographs of the CoCrFeMnNiAl*_x_* films with various Al contents: (**a**) Al_0_, (**b**) Al_0.07_, (**c**) Al_0.3_, (**d**) Al_0.6_, (**e**) Al_1.0_ and (**f**) Al_1.3_.

The microstructures of CoCrFeMnNiAl*_x_* HEAFs with various Al contents are presented in Figure 4. All films showed grain-like structures. The grain size of the film became larger with the increasing sputtering power. The higher sputtering power in the film deposition could offer higher translational kinetic energy to the adatoms and it could lead to an increase in the grain size [44,45]. In the duplex FCC + BCC phases region, however, there were many interphase boundaries which could result in the grain size refinement [46]. As a result, the grain size decreased when the Al content increased to Al_0.3_. With the further addition of Al to Al_1.0_, the structure transformed to a single BCC phase and the grain size of the film increased with the reduction in interphase boundaries [40]. In addition, some cracks could be observed along the grain boundaries and it might result from the internal stress during the film deposition [47]. Compared to CoCrFeMnNiAl*_x_* HEAs, CoCrFeMnNiAl*_x_* HEAF formed via rapid solidification due to the faster cooling rate [48]. A rapid cooling rate can limit the diffusion of the target elements; therefore, CoCrFeMnNiAl*_x_* HEAFs tend to inhibit the phase separation such as B2 AlNi−rich phase that usually formed in HEAs [17].

**Figure 5:**
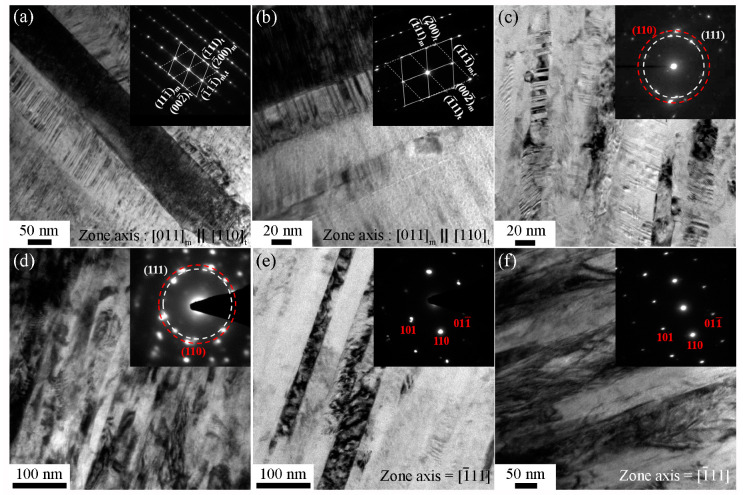
The cross-section TEM images and the corresponding SAED patterns of CoCrFeMnNiAl*_x_* films with different Al contents: (**a**) Al_0_, (**b**) Al_0.07_, (**c**) Al_0.3_, (**d**) Al_0.6_, (**e**) Al_1.0_ and (**f**) Al_1.3_.

A dual-beam focused ion beam was used to prepare the TEM samples, and the cross-section TEM images and the corresponding selected-area electron diffraction (SAED) patterns of CoCrFeMnNiAl*_x_* films with different Al contents are shown in Figure 5. All cross-section TEM images exhibit a columnar structure normal to the substrate. The TEM images of columnar grains with nanotwins are shown in Figure 5a,b, respectively, for Al_0_ and Al_0.07_. In order to reduce the strain energy, which might result from the release of residual stresses or thermal expansion difference between Si substrate and the film, twinning often appeared in films [49,50]. However, the stacking fault energy of Al is much higher than that of CoCrFeMnNi HEAs (~166 vs. ~25 mJ/m^2^) [51,52]. With the addition of Al, the stacking fault energy of the alloy would increase and twinning would not occur easily in BCC metals [40,53]. As a result, Al_1.0_ and Al_1.3_ showed only a single BCC structure and no twinning was observed. Also, while a small amount of nanotwins still could be observed in Al_0.3_, nanotwins could not be found in Al_0.6_, which was dominated by the BCC structure. The corresponding SAED patterns showed that the films had a phase transition from FCC for Al_0_ and Al_0.07_ to duplex phases (FCC + BCC) in Al_0.3_ and Al_0.6_ and to BCC phase in Al_1.0_ and Al_1.3_, which was consistent with the XRD results shown in Figure 1 and Figure 2.

**Figure 6:**
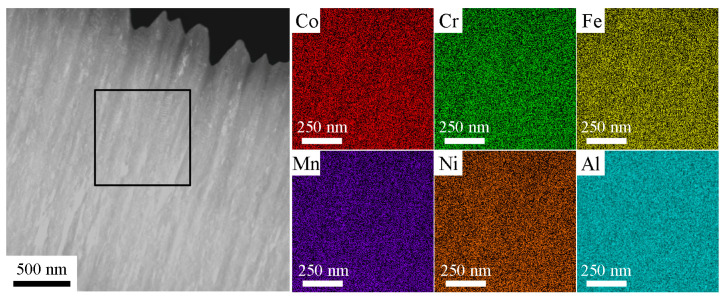
The cross-section TEM image and EDS mapping of the Al_0.3_ film.

The TEM−EDS mapping was used to analyze the elemental distribution of Al_0.3_ (with the duplex phases of FCC + BCC), as shown in Figure 6. There was a uniform distribution of the constituent elements (Co, Cr, Fe, Mn, Ni and Al) and no precipitates were found in the films. Therefore, it could be concluded from Figure 6 that the elemental distributions of Al_0.3_ films were homogeneous. The homogeneity of the elemental distribution was also observed in other CoCrFeMnNiAl*_x_* HEAFs.

### 3.3. Mechanical Properties

**Figure 7:**
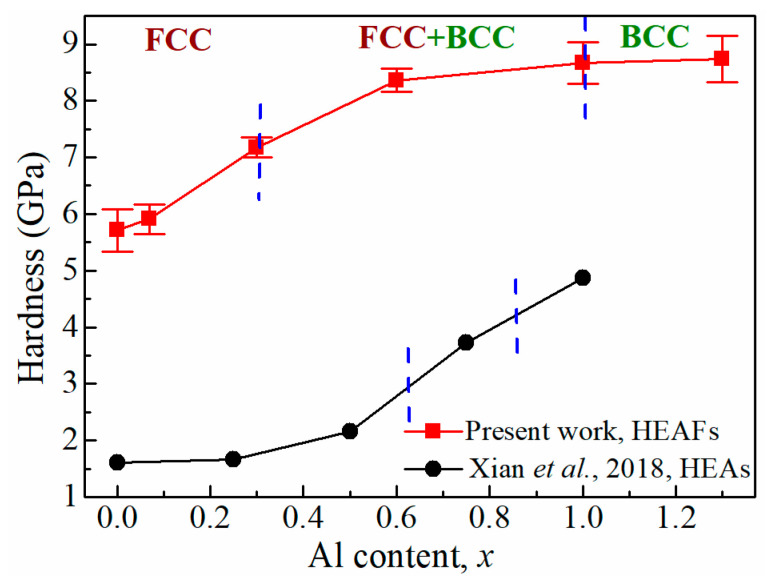
The hardnesses of CoCrFeMnNiAl*_x_* HEAFs and HEAs as functions of the Al content. The phase transformation from FCC to BCC is also indicated.

The hardness values of CoCrFeMnNiAl*_x_* films measured by nanoindentation are shown in Figure 7. The as-deposited CoCrFeMnNi film (Al_0_) exhibited a single FCC structure with a lower hardness of around 5.71 GPa, and the addition of a small amount of Al atoms resulted in an increase to 5.91 GPa in the FCC structure of Al_0.07_. With the further addition of Al, the hardness increased drastically to 8.36 GPa in the duplex FCC + BCC phases region. When the phase transformed to a single BCC structure, the Al_1.3_ film reached a maximum hardness of 8.74 GPa. As a result, the structural transition from FCC to BCC led to the hardness enhancements with the increasing Al content. It is worth noting that Al-doped CoCrFeMnNi HEAs have been processed and their mechanical properties have been characterized by Xian et al. [17] and the measured hardness values are also included in Figure 7 for comparison. Compared to Al-doped CoCrFeMnNi HEAs, Al-doped CoCrFeMnNi HEAFs processed in the present work had a much higher hardness, which could be attributed to the much smaller grain size of HEAFs (nm vs. μm).

**Figure 8:**
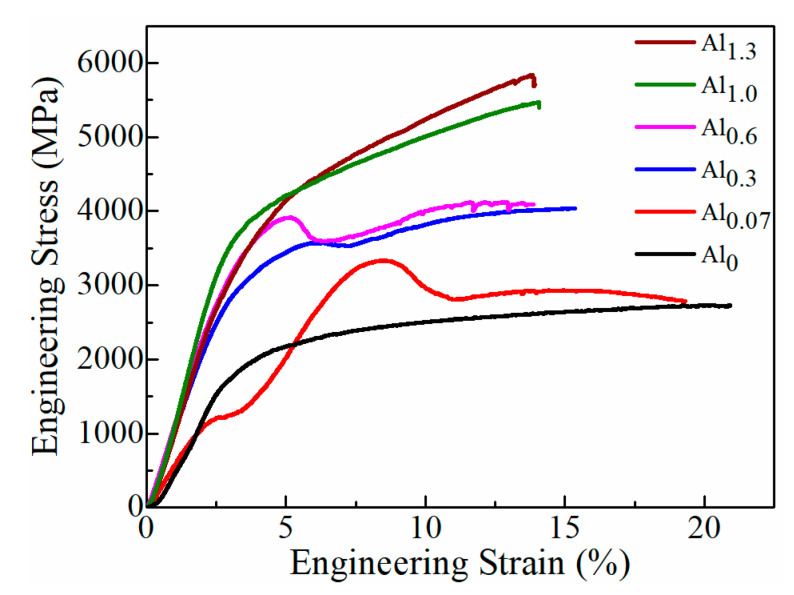
The compressive engineering stress−strain curves of the CoCrFeMnNiAl*_x_* HEAFs.

Using micropillar compression tests, the compressive stress−strain curves of CoCrFeMnNiAl*_x_* HEAFs are shown in Figure 8. A load drop was observed in Al_0.07_, Al_0.3_ and Al_0.6_ in the presence of the FCC phase and Al but not in Al_1.0_ and Al_1.3_, which had a single BCC phase. Because no precipitates were found in our films, we believed that the load drop could result from the pinning effect due to dislocation slip blocked by the solute atoms, and a higher stress was needed for dislocations to overcome the activation energy barrier. When the dislocations started to move away from the solute atoms, the loading stress dropped to a lower value and the pinning energy was released. The compressive yield strength, fracture strength and fracture strain are summarized in Table 2. The yield strengths of the Al_0_ and Al_0.07_ films were 1.59 and 3.32 GPa, respectively, and the values of the fracture strain were more than 19%. When the Al content increased to Al_0.6_, the yield strength and fracture strength increased to 3.91 and 4.16 GPa, respectively, while the fracture strain decreased to 13.86%. This was due to the formation of the BCC structure and a similar phenomenon was also observed in CoCrFeMnNiAl*_x_* HEAs [17]. When the phase transformed to a single BCC phase, the yield strength of the Al_1.3_ HEAF decreased to 3.73 GPa and the fracture strength reached a maximum value of 5.83 GPa. At the same time, the fracture strain decreased to 13.78%. As a result, it could be concluded from Figure 8 that both values of the yield strength and fracture strength increased, while the fracture strain decreased with the increasing Al content. These indicated that the addition of Al had a positive effect on enhancing the mechanical properties of the CoCrFeMnNi HEAFs.

## 4. Conclusions

CoCrFeMnNiAl*_x_* (*x* = 0, 0.07, 0.3, 0.6, 1.0, 1.3) HEAFs were successfully deposited by RF magnetron co-sputtering. The crystalline structure was verified by both XRD and TEM. The XRD results indicated the phase transformation from FCC to BCC with the increasing Al content. Also, the corresponding SAED patterns proved the phase transition from FCC to duplex phases (FCC + BCC) in Al_0.6_ and to BCC phase in Al_1.0_. The EDS element mapping showed that the elemental distributions in CoCrFeMnNiAl*_x_* HEAFs were homogeneous. Nanoindentation tests showed that the hardness increased from 5.71 GPa in Al_0_ to 8.74 GPa in Al_1.3_. The structural transition from FCC to BCC led to an increase in the hardness with the increasing Al content. Moreover, the mechanical properties of the films were measured using micropillar compression tests. The yield strength increased from 1.59 GPa in Al_0_ to 3.91 GPa in Al_0.6_, while the fracture strength reached a maximum value of 5.83 GPa in Al_1.3_. Meanwhile, the fracture strain decreased to 13.78% due to the formation of a harder BCC phase. Therefore, the nanoindentation measurements and the micropillar compression tests indicated that the addition of Al could strengthen the CoCrFeMnNi HEAFs.

## Figures and Tables

**Table 1:** The molar ratios and chemical compositions of CoCrFeMnNiAl*_x_* HEAFs with different powers applied on the Al target.

Power	*x*	Co	Cr	Fe	Mn	Ni	Al
0	0	19.77	18.39	19.97	22.80	19.07	0
10	0.07	19.34	18.20	19.86	22.61	18.65	1.34
20	0.3	19.48	18.24	19.76	17.69	18.79	6.04
40	0.6	18.18	17.94	17.83	18.37	16.78	10.90
60	1.0	16.55	15.49	16.68	17.90	15.75	17.63
80	1.3	15.92	14.89	16.39	17.42	15.45	19.93

**Table 2:** The compressive yield strength (*σ_y_*), fracture strength (*σ_f_*) and fracture strain (*ε_t_*) of CoCrFeMnNiAl*_x_* HEAFs.

*x*	*σ_y_* (GPa)	*σ_f_* (GPa)	*ε_t_* (%)
0	1.59	Not fractured	>20.91
0.07	3.32	Not fractured	>19.31
0.3	3.57	4.04	15.37
0.6	3.91	4.16	13.86
1.0	3.61	5.47	13.80
1.3	3.73	5.83	13.78

## References

[1] Yeh J.W., Chen S.K., Lin S.J., Gan J.Y., Chin T.S., Shun T.T., Tsau S.Y., Chang C.H. (2004). Nanostructured high-entropy alloys with multiple principal elements: Novel alloy design concepts and outcomes. Adv. Eng. Mater..

[2] Joo S.H., Kato H., Jang M.J., Moon J., Kim E.B., Hong S.J., Kim H.S. (2017). Structure and properties of ultrafine-grained CoCrFeMnNi high-entropy alloys produced by mechanical alloying and spark plasma sintering. J. Alloys Compd..

[3] Cantor B., Chang I.T.H., Knight P., Vincent A.J.B. (2004). Microstructural development in equiatomic multicomponent alloys. Mater. Sci. Eng. A.

[4] Cheng C.Y., Yeh J.W. (2016). High-entropy BNbTaTiZr thin film with excellent thermal stability of amorphous structure and its electrical properties. Mater. Lett..

[5] Sheng W., Yang X., Wang C., Zhang Y. (2016). Nano-crystallization of high-entropy amorphous NbTiAlSiW*_x_*N*_y_* films prepared by magnetron sputtering. Entropy.

[6] Tang Z., Huang L., He W., Liaw P.K. (2014). Alloying and processing effects on the aqueous corrosion behavior of high-entropy alloys. Entropy.

[7] Luo H., Li Z., Mingers A.M., Raabe D. (2018). Corrosion behavior of an equiatomic CoCrFeMnNi high-entropy alloy compared with 304 stainless steel in sulfuric acid solution. Corros. Sci..

[8] Hsu C.Y., Sheu T.S., Yeh J.W., Chen S.K. (2010). Effect of iron content on wear behavior of AlCoCrFe*_x_*Mo_0.5_Ni high-entropy alloys. Wear.

[9] Ye Y.X., Liu C.Z., Wang H., Nieh T.G. (2018). Friction and wear behavior of a single-phase equiatomic TiZrHfNb high-entropy alloy studied using a nanoscratch technique. Acta Mater..

[10] Zhang Y., Zuo T.T., Tang Z., Gao M.C., Dahmen K.A., Liaw P.K., Lu Z.P. (2014). Microstructures and properties of high-entropy alloys. Prog. Mater. Sci..

[11] Wang C., Li T.H., Liao Y.C., Li C.L., Jang J.S.C., Hsueh C.H. (2019). Hardness and strength enhancements of CoCrFeMnNi high-entropy alloy with Nd doping. Mater. Sci. Eng. A.

[12] Otto F., Dlouhý A., Somsen C., Bei H., Eggeler G., George E.P. (2013). The influences of temperature and microstructure on the tensile properties of a CoCrFeMnNi high-entropy alloy. Acta Mater..

[13] Schuh B., Mendez-Martin F., Völker B., George E.P., Clemens H., Pippan R., Hohenwarter A. (2015). Mechanical properties, microstructure and thermal stability of a nanocrystalline CoCrFeMnNi high-entropy alloy after severe plastic deformation. Acta Mater..

[14] He J.Y., Wang H., Huang H.L., Xu X.D., Chen M.W., Wu Y., Liu X.J., Nieh T.G., An K., Lu Z.P. (2016). A precipitation-hardened high-entropy alloy with outstanding tensile properties. Acta Mater..

[15] Gludovatz B., Hohenwarter A., Catoor D., Chang E.H., George E.P., Ritchie R.O. (2014). A fracture-resistant high-entropy alloy for cryogenic applications. Science.

[16] He J.Y., Liu W.H., Wang H., Wu Y., Liu X.J., Nieh T.G., Lu Z.P. (2014). Effects of Al addition on structural evolution and tensile properties of the FeCoNiCrMn high-entropy alloy system. Acta Mater..

[17] Xian X., Zhong Z.H., Lin L.J., Zhu Z.X., Chen C., Wu Y.C. (2018). Tailoring strength and ductility of high-entropy CrMnFeCoNi alloy by adding Al. Rare Met..

[18] Kumar J., Kumar N., Das S., Gurao N.P., Biswas K. (2018). Effect of Al addition on the microstructural evolution of equiatomic CoCrFeMnNi alloy. Trans. Indian Inst. Met..

[19] Wu C.S., Tsai P.H., Kuo C.M., Tsai C.W. (2018). Effect of Atomic Size Difference on the Microstructure and Mechanical Properties of High-Entropy Alloys. Entropy.

[20] Wang W.R., Wang W.L., Wang S.C., Tsai Y.C., Lai C.H., Yeh J.W. (2012). Effects of Al addition on the microstructure and mechanical property of Al*_x_*CoCrFeNi high-entropy alloys. Intermetallics.

[21] Xia S.Q., Gao M.C., Zhang Y. (2018). Abnormal temperature dependence of impact toughness in Al*_x_*CoCrFeNi system high entropy alloys. Mater. Chem. Phys..

[22] Rao J.C., Ocelík V., Vainchtein D., Tang Z., Liaw P.K., De Hosson J.T.M. (2016). The fcc-bcc crystallographic orientation relationship in Al*_x_*CoCrFeNi high-entropy alloys. Mater. Lett..

[23] Yang T., Xia S., Liu S., Wang C., Liu S., Zhang Y., Xue J., Yan S., Wang Y. (2015). Effects of Al addition on microstructure and mechanical properties of Al*_x_*CoCrFeNi High-entropy alloy. Mater. Sci. Eng. A.

[24] Guo S., Ng C., Lu J., Liu C.T. (2011). Effect of valence electron concentration on stability of fcc or bcc phase in high entropy alloys. J. Appl. Phys..

[25] Tong C.J., Chen Y.L., Yeh J.W., Lin S.J., Chen S.K., Shun T.T., Tsau C.H., Chang S.Y. (2005). Microstructure characterization of Al*_x_*CoCrCuFeNi high-entropy alloy system with multiprincipal elements. Metall. Mater. Trans. A.

[26] Wu J.M., Lin S.J., Yeh J.W., Chen S.K., Huang Y.S., Chen H.C. (2006). Adhesive wear behavior of Al*_x_*CoCrCuFeNi high-entropy alloys as a function of aluminum content. Wear.

[27] Mao A., Ding P., Quan F., Zhang T., Ran X., Li Y., Jin X., Gu X. (2018). Effect of aluminum element on microstructure evolution and properties of multicomponent Al-Co-Cr-Cu-Fe-Ni nanoparticles. J. Alloys Compd..

[28] Zhang K., Fu Z. (2012). Effects of annealing treatment on phase composition and microstructure of CoCrFeNiTiAl*_x_* high-entropy alloys. Intermetallics.

[29] Zhou Y.J., Zhang Y., Wang Y.L., Chen G.L. (2007). Microstructure and compressive properties of multicomponent Al*_x_*(TiVCrMnFeCoNiCu)_100−*x*_ high-entropy alloys. Mater. Sci. Eng. A.

[30] Kim W.J., Jeong H.T., Park H.K., Park K., Na T.W., Choi E. (2019). The effect of Al to high-temperature deformation mechanisms and processing maps of Al_0.5_CoCrFeMnNi high entropy alloy. J. Alloys Compd..

[31] Liu Y., Chen Z., Shi J., Wang Z., Zhang J. (2019). The effect of Al content on microstructures and comprehensive properties in Al*_x_*CoCrCuFeNi high entropy alloys. Vacuum.

[32] Brechtl J., Chen S.Y., Xie X., Ren Y., Qiao J.W., Liaw P.K., Zinkle S.J. (2019). Towards a greater understanding of serrated flows in an Al-containing high-entropy-based alloy. Int. J. Plast..

[33] Chen S., Xie X., Li W., Feng R., Chen B., Qiao J., Ren Y., Zhang Y., Dahmen K.A., Liaw P.K. (2018). Temperature effects on the serrated behavior of an Al_0.5_CoCrCuFeNi high-entropy alloy. Mater. Chem. Phys..

[34] Niu S., Kou H., Zhang Y., Wang J., Li J. (2017). The characteristics of serration in Al_0.5_CoCrFeNi high entropy alloy. Mater. Sci. Eng. A.

[35] Greer A., Cheng Y., Ma E. (2013). Shear bands in metallic glasses. Mater. Sci. Eng. R Rep..

[36] Chen C.S., Yiu P., Li C.L., Chu J.P., Shek C.H., Hsueh C.H. (2014). Effects of annealing on mechanical behavior of Zr–Ti–Ni thin film metallic glasses. Mater. Sci. Eng. A.

[37] Wu Y.H., Wang C., Hsueh C.H., Li T.H., Chang C.H., Chen H.C., Jang J.S.C., Huang J.C., Ma Z.H. (2017). Microstructure and mechanical properties of Zr-Ti-Cu-Nd metallic glass composites. J. Alloys Compd..

[38] Bizhanova G., Li F., Ma Y., Gong P., Wang X. (2019). Development and crystallization kinetics of novel near-equiatomic high-entropy bulk metallic glasses. J. Alloys Compd..

[39] Gao L., Liao W., Zhang H., Surjadi J., Sun D., Lu Y. (2017). Microstructure, mechanical and corrosion behaviors of CoCrFeNiAl_0.3_ high entropy alloy (HEA) films. Coatings.

[40] Feng X.B., Fu W., Zhang J.Y., Zhao J.T., Li J., Wu K., Liu G., Sun J. (2017). Effects of nanotwins on the mechanical properties of Al*_x_*CoCrFeNi high entropy alloy thin films. Scr. Mater..

[41] Sun X., Zhang H., Lu S., Ding X., Wang Y., Vitos L. (2017). Phase selection rule for Al-doped CrMnFeCoNi high-entropy alloys from first-principles. Acta Mater..

[42] Yang X., Zhang Y. (2012). Prediction of high-entropy stabilized solid-solution in multi-component alloys. Mater. Chem. Phys..

[43] Sheng G., Liu C.T. (2011). Phase stability in high entropy alloys: Formation of solid-solution phase or amorphous phase. Prog. Nat. Sci. Mater. Int..

[44] Chan K.Y., Teo B.S. (2007). Investigation into the influence of direct current (DC) power in the magnetron sputtering process on the copper crystallite size. Microelectron. J..

[45] Andújar J., Pino F., Polo M., Pinyol A., Corbella C., Bertran E. (2002). Effects of gas pressure and rf power on the growth and properties of magnetron sputter deposited amorphous carbon thin films. Diam. Relat. Mater..

[46] Jo Y., Choi W., Kim D., Zargaran A., Sohn S.S., Kim H.S., Lee B., Kim N.J., Lee S. (2019). FCC to BCC transformation-induced plasticity based on thermodynamic phase stability in novel V_10_Cr_10_Fe_45_Co*_x_*Ni_35−*x*_ medium-entropy alloys. Sci. Rep..

[47] Tsau C.H., Chang Y.H. (2013). Microstructures and mechanical properties of TiCrZrNbN*_x_* alloy nitride thin films. Entropy.

[48] Li W., Liu P., Liaw P.K. (2018). Microstructures and properties of high-entropy alloy films and coatings: A review. Mater. Res. Lett..

[49] Sridhar N., Rickman J.M., Srolovitz D.J. (1996). Twinning in thin films—I. Elastic analysis. Acta Mater..

[50] Sridhar N., Rickman J.M., Srolovitz D.J. (1996). Twinning in thin films—II. Equilibrium microstructures. Acta Mater..

[51] Velasco L., Hodge A.M. (2016). The mobility of growth twins synthesized by sputtering: Tailoring the twin thickness. Acta Mater..

[52] Zaddach A.J., Niu C., Koch C.C., Irving D.L. (2013). Mechanical properties and stacking fault energies of NiFeCrCoMn high-entropy alloy. JOM.

[53] Wang J., Zeng Z., Weinberger C.R., Zhang Z., Zhu T., Mao S.X. (2015). In situ atomic-scale observation of twinning-dominated deformation in nanoscale body-centred cubic tungsten. Nat. Mater..

